# A Preliminary Study of a Lettuce-Based Edible Vaccine Expressing the Cysteine Proteinase of *Fasciola hepatica* for Fasciolosis Control in Livestock

**DOI:** 10.3389/fimmu.2018.02592

**Published:** 2018-11-13

**Authors:** Agnieszka Wesołowska, Monika Kozak Ljunggren, Luiza Jedlina, Katarzyna Basałaj, Andrzej Legocki, Halina Wedrychowicz, Małgorzata Kesik-Brodacka

**Affiliations:** ^1^Polish Academy of Sciences, Witold Stefanski Institute of Parasitology, Warsaw, Poland; ^2^Polish Academy of Sciences, Institute of Bioorganic Chemistry, Poznan, Poland; ^3^Department of Bioengineering, Institute of Biotechnology and Antibiotics, Warsaw, Poland

**Keywords:** lettuce-derived vaccine, oral delivery, *Fasciola hepatica*, ruminants, cysteine protease

## Abstract

Oral vaccination with edible vaccines is one of the most promising approaches in modern vaccinology. Edible vaccines are an alternative to conventional vaccines, which are typically delivered by injection. Here, freeze-dried transgenic lettuce expressing the cysteine proteinase of the trematode *Fasciola hepatica* (CPFhW) was used to orally vaccinate cattle and sheep against fasciolosis, which is the most important trematode disease due to the parasite's global distribution, wide spectrum of host species and significant economic losses of farmers. In the study, goals such as reducing the intensity of infection, liver damage and *F. hepatica* fecundity were achieved. Moreover, we demonstrated that the host sex influenced the outcome of infection following vaccination, with female calves and male lambs showing better protection than their counterparts. Since differences occurred following vaccination and infection, different immunization strategies should be considered for different sexes and host species when developing new control methods. The results of the present study highlight the potential of oral vaccination with plant-made and plant-delivered vaccines for *F. hepatica* infection control.

## Introduction

Fasciolosis is a chronic disease with a global distribution. This disease is a major cause of morbidity and mortality in domestic ruminants, such as cattle and sheep, and results in an estimated loss of approximately US$3 billion annually to the global agricultural sector (1). Drugs may provide a cure for liver fluke infection; however, due to the continuous development of drug-resistant parasites and high reinfection rates in areas where *Fasciola hepatica* exposure is a regular occurrence, new control strategies against fasciolosis are needed. Thus, efforts have been made to develop vaccines capable of providing protection in vaccinated animals of economic importance, although no commercially available vaccine against ovine or bovine fasciolosis is available at present. Many vaccine studies in ruminants using different candidate antigens, including the leading candidates fatty acid binding protein, glutathione-s-transferase, leucine aminopeptidase, and cathepsin (Cat) L1 and L2, have shown promise (2). In particular, vaccine preparations containing cathepsins are the most promising (3). Native CatL1 has shown up to 69% protection in cattle (4), and CatL1 mimotopes induce up to 79% protection in goats (5). Furthermore, a trivalent vaccine containing CatL1 and CatL2 combined with leucine aminopeptidase (LAP) has shown significant efficacy of 79% in sheep (6). Although all of these vaccines are administered by injection, effective non-parenteral vaccines have also been tested in ruminants. For instance, the intranasal and oral delivery of CPFhW CatL in the form of inclusion bodies showed 54 and 61% protection in cattle and sheep, respectively (7).

Recently, the oral vaccine delivery route has received increasing attention due to its proven potential for veterinary use (8, 9). The edible vaccine concept refers to oral immunization with antigens expressed in recombinant plant tissues. Since many pathogens invade their hosts through mucosal surfaces, such as the gastrointestinal mucosa, the generation of a vaccine capable of inducing protective immune responses at the parasite entry site is a very attractive strategy. Plant-based vaccines can effectively stimulate humoral and cellular responses at both mucosal and systemic sites, thereby providing effector arms to achieve protection. Another major advantage of edible plant-derived vaccines is their easy application for oral delivery. Antigens expressed in an edible plant may be used as an oral vaccine without processing, including the expensive purification steps that are generally required for parenterally administered vaccines (10). Moreover, the use of plants to produce pathogen antigens ensures that all post-translational modifications are completed in the protein of interest, since plants possess the expression, folding, assembly, and glycosylation machinery needed to achieve the antigen's structure and biological activity. Edible vaccines are also attractive in terms of safety, because they lack animal or human pathogens. Additionally, eliminating some of the complicated downstream processing steps diminishes the overall vaccine production cost. Plant-based edible vaccines are a cold chain-free, needle-free, and potentially economically viable intervention strategy against infectious diseases.

Substantial protection has been obtained in rats after oral vaccination with lyophilized transgenic lettuce expressing CPFhW CatL fused to a hepatitis B virus core antigen (HBcAg) carrier (up to a 65.5% reduction in the liver fluke burden) (11). Here, we investigated the potential of a lettuce-based edible vaccine expressing CPFhW fused to HBcAg against subsequent infection with liver fluke metacercariae in the natural hosts of *F. hepatica* (sheep and cattle).

## Materials and methods

### Ethics statement

All experimental procedures were approved by the III Local Animal Experimentation Ethics Committee, Warsaw, Poland (approval number 39/2003), and were performed according to the guidelines of the European Communities Council Directive (86/609/EEC). All efforts were made to minimize animal suffering and to reduce the number of animals used.

### Vaccine construct

The vaccine construct was obtained as previously described (11). Briefly, cDNA encoding CPFhW (accession no. AY277628) cloned into the pcDNA3.1 plasmid was used to amplify the sequence encoding the mature CPFhW. The HBV 321 plasmid provided the sequence of the entire hepatitis B virus (HBV, *ayw4*) genome for amplification of the region encoding the truncated core HBV protein (HBcAg(T); accession no. Z35716). The construct encoding the fusion protein HBcAg(T) with an insertion encoding CPFhW flanked by Gly-rich linkers (mCPFhW::G::C) was created and placed in a pROK2 plant expression vector prior to transformation of the *Agrobacterium tumefaciens* LBA 4404 strain.

### Transgenic lettuce

The transgenic lettuce expressing the vaccine antigen was obtained as previously described (12). Briefly, the *A. tumefaciens* LBA 4404 strain transformed with the ROK2 expression vector encoding HBcAg(T) with the CPFhW insertion flanked by Gly-rich linkers was used to transform lettuce (*Lactuca sativa*). Leaves from the transgenic lettuce were lyophilized prior to being fed to the animals, and the amount of vaccine antigen was calculated based on quantitative enzyme-linked immunosorbent assay (ELISA) results. A Microtiter MaxiSorp (NUNC, Denmark) microplate was coated with anti-CPFhW mouse IgG1 monoclonal antibody (antibodies used in ELISA were produced in Institute of Biotechnology and Antibiotics against CPFhW) (1 μg/ml in carbonate buffer, pH 9.6). The plate was incubated for 3 h at 37°C and subsequently washed tree times with phosphate buffer (PBS) pH 7.4 containing 0.05% Tween 20. The coated plate was saturated with 5% (w/v) PBS-fat-free milk at 25°C for 1 h. Plant extracts were serially diluted in PBS. Incubation with extract was conducted overnight at 4°C. Following three washes, wells were incubated with 1:5,000 anti-CPFhW rabbit polyclonal antibody (produced in Institute of Biotechnology and Antibiotics against CPFhW) at 37°C for 1.5 h, then plates were washed three times and incubated with 1:20,000 anti-rabbit IgG alkaline phosphatase-conjugated mAb (Sigma). The reaction with p-nitrophenylphosphate as the substrate in 2-amino-2-methyl-1,3-propanediol buffer (ICN) was developed at 25°C for 1 h and absorbance was measured at 405 nm using a Microplate Reader, Model 550 (Bio-Rad, USA). The antigen concentration was calculated in micrograms per gram of lyophilised mass using a standard curve utilizing known concentrations of recombinant CPFhW.

### Parasites

The Weybridge strain of *F. hepatica* that was maintained in our laboratory was used for the cattle and sheep infections (13). Metacercariae were obtained as previously described (11), and their viability was tested prior to infection. Two-month-old metacercariae were used for the infection experiments.

### Vaccination trial

Twelve Corriedale lambs and twelve Holstein-Friesian calves were purchased from a fluke-free area. The animals were shown to be free of infection by fecal analysis and ELISA using *F. hepatica* excretory-secretory material. During the experiment, the animals were housed indoors and fed hay and pelleted nutritional concentrate. Water was available *ad libitum*. The lambs and calves were 5 months old and 5–7 months old at the beginning of the experiment, respectively.

The cattle were allocated randomly into two groups of 6 animals that contained 3 males and 3 females each. The same scheme was applied to the sheep. The calves and lambs were orally administered either a suspension of freeze-dried transgenic lettuce containing 500 or 300 μg of the mCPFhW::G::C protein, respectively, or the same amount of freeze-dried unmodified lettuce (control group). The animals were fed the same doses of transgenic or unmodified lettuce twice at 4-week intervals. Four weeks after the second immunization, the calves and lambs were orally challenged with a gelatine capsule containing 400 or 250 metacercariae, respectively. The animals were slaughtered 12 weeks post-infection (WPI) and necropsied.

### Sampling

Fecal samples were collected from each animal prior to vaccination and infection and then every week starting from 6 WPI. The fecal samples were examined using the sedimentation method (14).

Blood samples were collected by jugular venepuncture from each animal prior to the first immunization and then biweekly until the time of slaughter. Serum was separated and stored at −70°C prior to use.

### Hematology and liver enzyme analysis

Blood samples were subjected to analysis in an automated analyser (Abacus JunVet) to monitor the total white blood cell count (WBC), lymphocyte, neutrophil, and eosinophil counts and red cell-related parameters during the study.

Liver tissue damage was assessed in all vaccinated and control animals by measuring the lactate dehydrogenase (LDH; EC 1.1.1.27) and gamma-glutamyl transferase (GGT; EC 2.3.2.2) activities in the blood samples using commercial kits according to the manufacturer's instructions (Boehringer). The results are expressed in International Units per liter (IU/l).

### ELISA test

Antibody responses were analyzed using ELISA. The wells of polystyrene microplates (Nunc, Roskilde, Denmark) were coated with a 5 μl/ml solution of recombinant CPFhW in carbonate buffer (pH 9.6) and incubated overnight at 4°C. The remaining binding sites were blocked with a 5% solution of soya milk in PBS for 1 h at 37°C; then, the plate wells were washed three times in 0.05% Tween 20 in PBS. Sera from individual animals were serially diluted along the plates starting from a 1:50 dilution. After 1 h of incubation at 37°C, the plates were washed as described above, and bound antibodies were detected with peroxidase-conjugated anti-ovine IgG or anti-bovine IgG and anti-ovine IgA or anti-bovine IgA (Bethyl Laboratories). After incubation for 1 h at 37°C, the plates were washed four times with 0.05% Tween 20 in PBS. The reaction was developed with a tetramethylbenzidine solution (Sigma), stopped with 2 M sulfuric acid and read at 450 nm on a spectrophotometer (HT Synergy, BIOTEK). The optimal concentrations of antigen (5 μl/ml) and antibodies (for IgG 1:100,000; for IgA 1:50,000) were determined by sequential titration using known positive and negative sera.

### Flow cytometry

Fifty microlitres of blood samples were transferred into cytometric tubes and incubated with antibodies directed against CD11a (HUH73A, IgG1), CD8α (7C2B, IgG2a), and CD4 (GC50A, IgM)(WSU Monoclonal Antibody Center, Pullman WA). All above mentioned antibodies have both bovine and ovine reactivity. Following 20 min incubation, cells were washed with phosphate buffered saline (PBS) and indirect labeling was performed by adding appropriate secondary rat anti-mouse antibodies conjugated with FITC, PE and APC (BD Pharmingen). After subsequent 20 min incubation excess of antibody was washed off with PBS. Erythrocytes were lysed with lysing solution (BD FACS) for 10 min. samples were washed twice and fixated with fixing solution (BD Cell Fix). Ten thousand gated events were counted in FACS Calibur flow cytometer and analyzed using CellQuest software.

### Necropsy and determination of protection

At necropsy, the livers and gall bladders were removed, and the index of liver damage was assigned a score of 0–5 according to the scale proposed by Raadsma et al. (15). Flukes found in the main bile duct and gall bladder were removed. The livers were cut into 1-cm-long pieces, soaked in water at 37°C for 30 min, squeezed, and forced through a 300-μm mesh sieve. The retained material was analyzed for immature or damaged flukes according to the process described in Ramajo et al. (16). The total number of flukes was counted, and the percent reduction was calculated according to the formula: P = (1 – V/C) × 100%, where V indicates the mean number of flukes in the vaccinated animals and C indicates the mean number of flukes in the control animals of a given species. Moreover, the number of eggs in the gall bladder was estimated using the quantitative sedimentation technique (14).

### Statistical analysis

Data was tested for normality (Shapiro–Wilk test) and variance homogeneity (Levene's test). The Mann–Whitney *U*-test was used to analyse the obtained data. The analyses were performed using Statistica 6.1 software. *p* < 0.05 were considered significant.

### Accession numbers

The genetic sequences used in this study were deposited in GenBank with accession numbers AY277628 for CPFhW and Z35716 for HBcAg.

## Results

### Fluke burden, fecundity, and viability

The liver fluke burdens reported in cattle and sheep are listed in Table 1. Vaccinated calves showed a significant reduction in liver fluke recovery of 56.2%. When the data were analyzed by sex, the female cattle were better protected than the males (68.1 and 45.8%, respectively; Table S1). For lambs, a 35.5% decrease in fluke numbers was observed; however, the difference was not significant. Only the male lambs had significantly lower worm burdens than the respective male challenge control group (54.7%). No significant differences in fluke burdens were found between the vaccinated female lambs and the control group (20.3%) (Table S1).

**Table 1:** Analysis of liver fluke recoveries in infected cattle and sheep at 12 WPI.

**Group**	**Liver fluke counts**	**Total liver fluke counts**	**Mean liver fluke count ±SD**	**Reduction in liver fluke burden**
Cattle fed with CPFhW/lettuce	16, 20, 24, 36, 39, 42	177	29.5 ± 10.9^*^	56.2%
Cattle fed with control lettuce	48, 56, 59, 59, 81, 101	404	67.0 ± 19.8	
Sheep fed with CPFhW/lettuce	25, 27, 36, 44, 80, 138	350	58.3 ± 43.8	35.5%
Sheep fed with control lettuce	65, 79, 84, 92, 94, 127	541	90.2 ± 20.8	

* *denotes a significant difference compared to respective control group (p < 0.05)*.

All animals had zero parasite eggs per gram of feces in the fecal material collected prior to the challenge infection. Fluke eggs were detected in the feces from 10 WPI. The differences in egg numbers per gram of feces between the experimental groups at 12 WPI are listed in Table 2. Significant differences were noted only in cattle fed the freeze-dried lettuce expressing CPFhW fused to HBcAg. An analogous analysis of the lambs revealed no differences between groups. Furthermore, an analysis of egg numbers in the gall bladders of the lambs showed a clear and significant reduction in the egg numbers (Table 2). Cattle vaccinated with transgenic lettuce expressing CPFhW fused to HBcAg also had lower fluke egg numbers than cattle receiving freeze-dried unmodified lettuce. Female calves had decreased fluke egg counts compared to the control group; this effect was not seen among the male cattle (Table 2). For sheep sex differences were not reported (Table S2).

**Table 2:** Numbers of fluke eggs found in the infected cattle and sheep of both sexes at necropsy (12 WPI).

**Group**	**No. of eggs per gram of feces**	**No. of fluke eggs in the gall bladders**
Cattle fed with CPFhW/lettuce	2.00 ± 0.89^*^	39,213 ± 16,663^*^
Cattle fed with control lettuce	4.00 ± 0.63	70,743 ± 13,019
Sheep fed with CPFhW/lettuce	2.50 ± 1.05	38,622 ± 31,894^*^
Sheep fed with control lettuce	3.80 ± 1.47	308,177 ± 217,375

* *Denotes a significant difference compared to respective control group (p < 0.05)*.

Vaccination affected the fluke body size in the cattle fed transgenic lettuce expressing CPFhW fused to HBcAg but not in the vaccinated sheep (Table 3). A shift toward a lower body size was observed in the vaccinated cattle compared to the control animals; this effect was more pronounced among the female cattle (Table S3).

**Table 3:** Vaccination influence on fluke body size.

**Group**	**Percentage [%] of flukes with body size [mm]**		
	**< 10**	**10–20**	**>20**
Cattle fed with CPFhW/lettuce	25	51	24
Cattle fed with control lettuce	11	65	24
Sheep fed with CPFhW/lettuce	21	61	18
Sheep fed with control lettuce	16	63	21

### Liver pathology

At necropsy, hepatic damage was scored 0–5 in the livers collected from the infected cattle and sheep. CPFhW-vaccinated cattle had decreased liver damage scores, with the lowest damage scores observed for females fed the transgenic lettuce expressing CPFhW fused to HBcAg. The vaccinated sheep also had lower liver damage scores than the challenge controls, but these differences were not significant (Table 4 and Table S4).

**Table 4:** Liver damage scores.

**Group**	**Liver damage scores**
Cattle fed with CPFhW/lettuce	2.67 ± 0.82
Cattle fed with control lettuce	3.83 ± 0.75
Sheep fed with CPFhW/lettuce	3.67 ± 0.52
Sheep fed with control lettuce	4.50 ± 0.55

**Figure 1:**
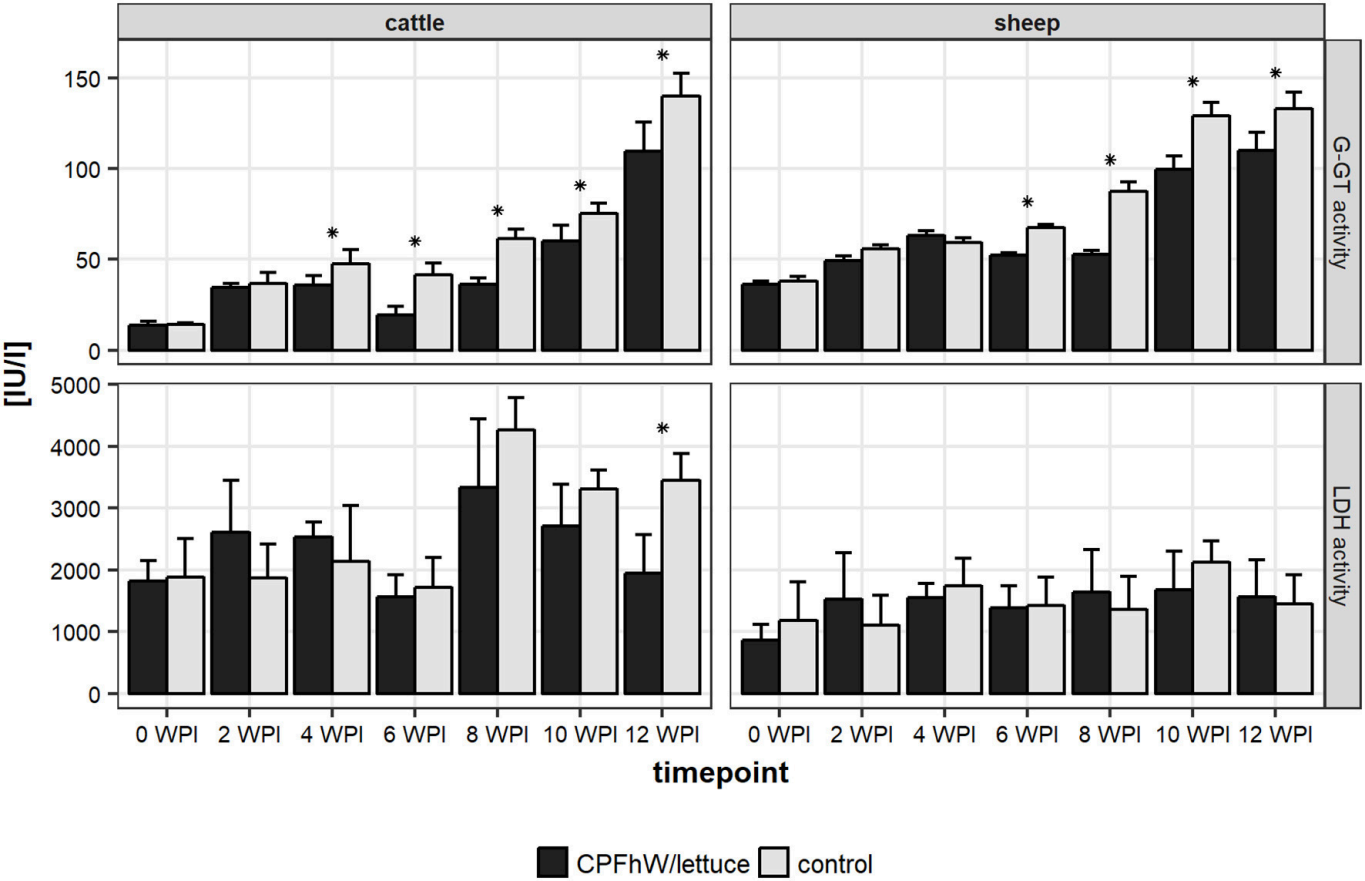
Liver enzymes activities in sera collected from experimental animals throughout the study. *Represents statistically significant differences (*p* < 0.05).

Changes in the hepatic enzyme levels that occurred during the experimental infections of cattle and sheep are shown in Figure 1 and Figure S1. Increased GGT activity was seen in the vaccinated and control groups from 2 WPI. For both the calves and lambs, significant decreases were observed in the vaccinated animals compared to the control groups from 4 and 6 WPI, respectively. For LDH activities no differences between vaccinated and control groups were observed.

### Cellular responses in the blood

**Figure 2:**
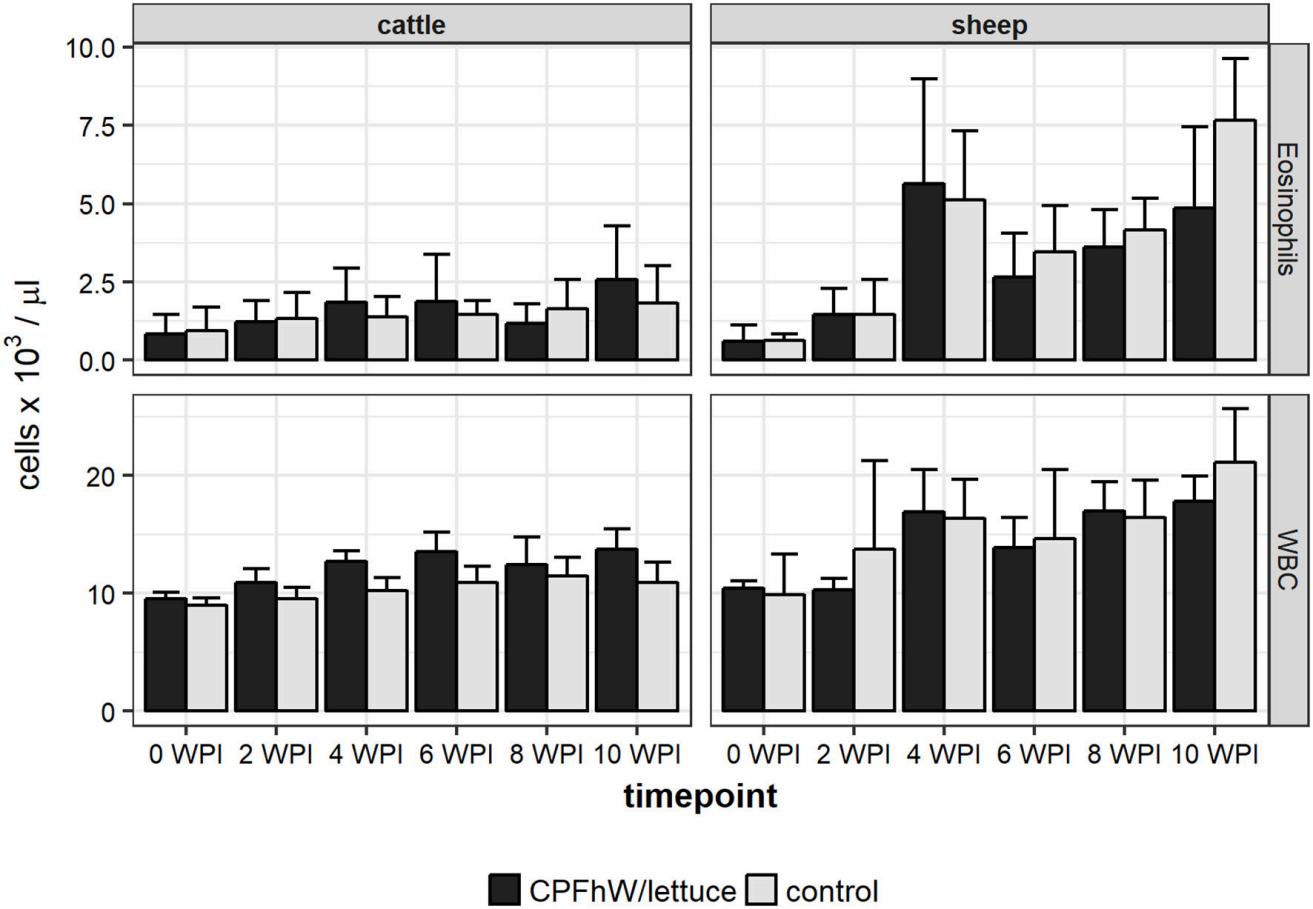
Eosinophil and total white blood cell (WBC) counts in blood samples collected from experimental animals.

No significant variations were observed in the blood neutrophil, monocyte or lymphocyte counts or the erythrocyte-related parameters (data not shown). When male and female cattle were compiled there were no statistically significant variations in eosinophil counts throughout the study (Figure 2). Only male calves vaccinated with transgenic lettuce had increased eosinophil level at 4 WPI (Figure S2). In sheep a sharp rise in eosinophil number was seen at 4 WPI (Figure 2).

### Flow cytometry

**Figure 3:**
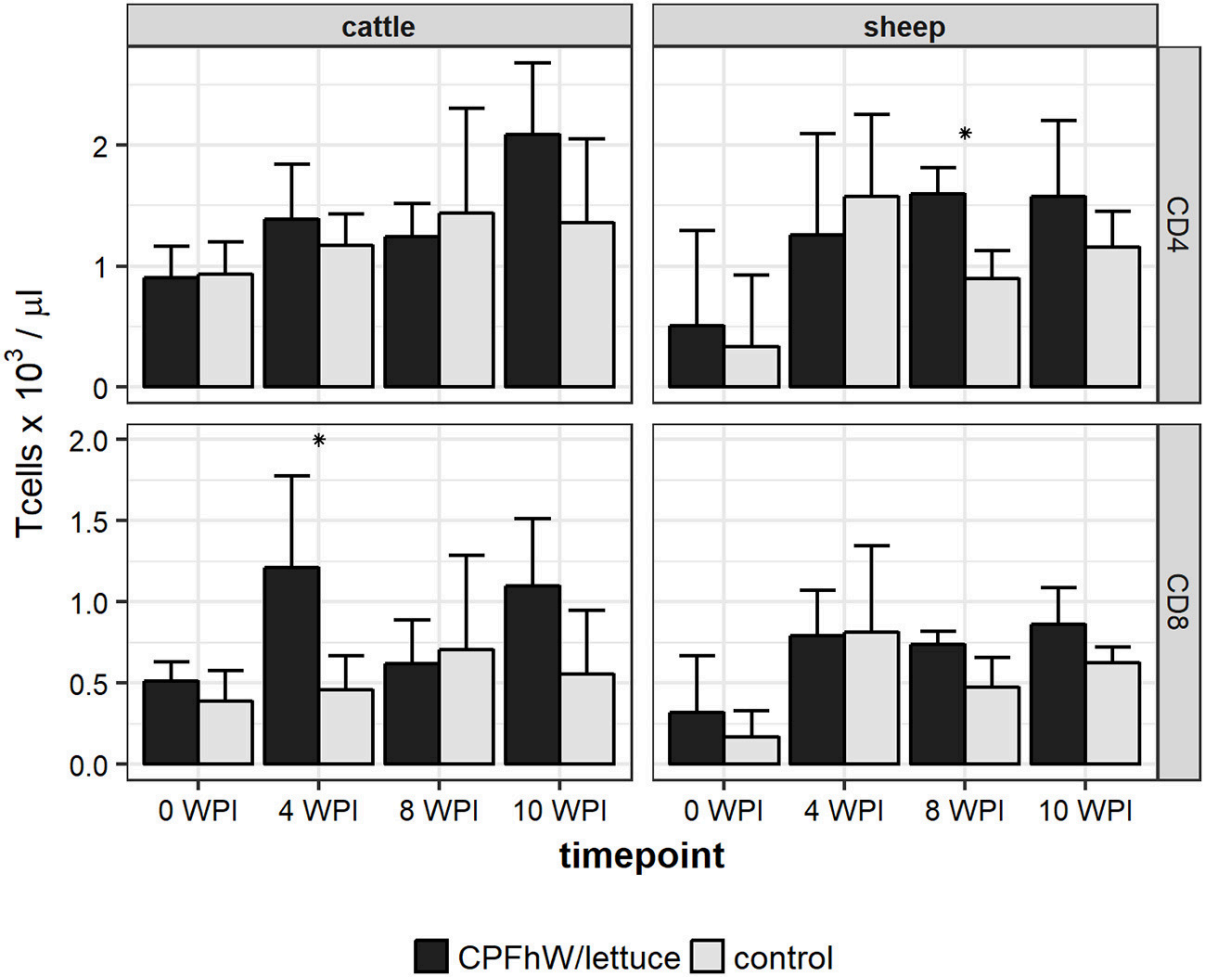
CD4 and CD8 T cell counts in blood samples collected from experimental animals. *Represents statistically significant differences (*p* < 0.05).

Flow cytometry analysis revealed that the only statistically significant difference in the count of CD4+ T cells was observed for sheep at 8 WPI, whereas the only statistically significant difference in the count of CD8+ T cells was reported for cattle at 4 WPI (Figure 3). When data was analyzed by sex, it was apparent that increase in CD8+ T cell count in vaccinated cattle was primarily attributed to vaccinated males not females (Figure S3).

### Serum antibody responses

**Figure 4:**
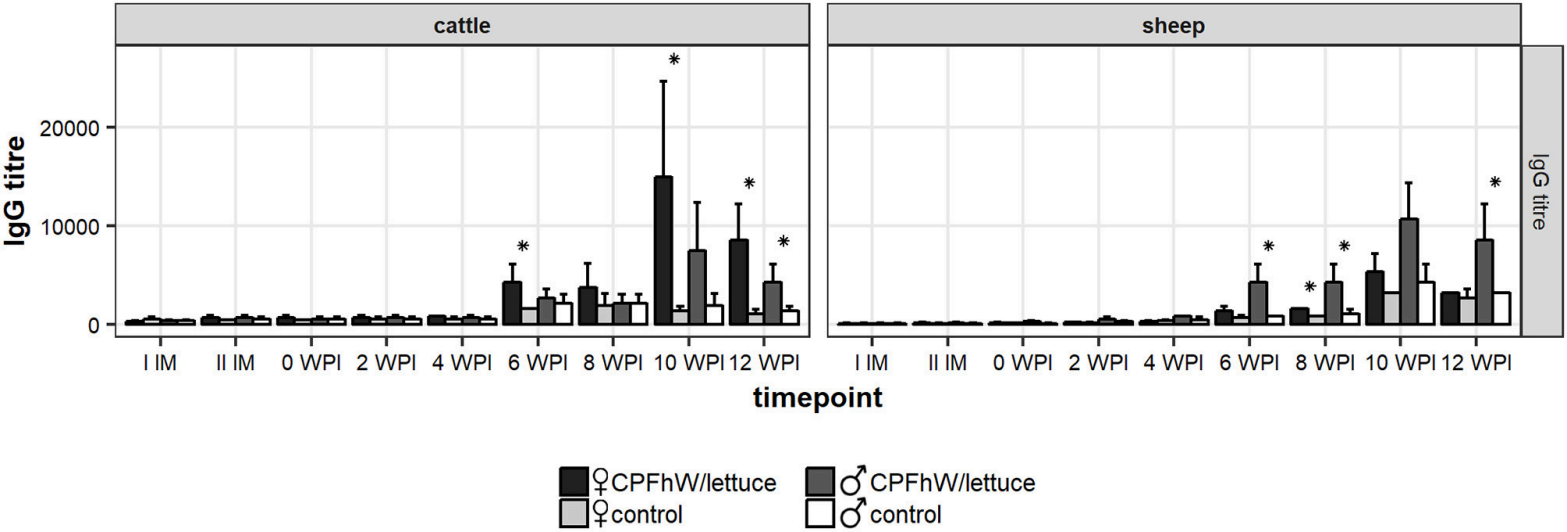
Total IgG levels in sera collected from experimental animals. *Represents statistically significant differences (*p* < 0.05).

The anti-CPFhW IgG response profiles of the calves and lambs are shown in Figure 4. Increased IgG levels were noted in the CPFhW-vaccinated animals of both species from 6 WPI and peaked at 10 WPI, whereas the levels observed for the controls remained low throughout the study. When the data were analyzed by sex, the female cattle had higher anti-CPFhW IgG levels from 6 WPI to the end of the experiment when compared with their male counterparts (Figure S4).

A sex-based analysis in the lambs demonstrated a significant increase in the IgG levels among the vaccinated males from 6 to 12 WPI. Among the female lambs, an increase in the IgG levels was seen during the same period; however, this increase was significant only when compared with the values reported on the day of challenge. When the data were compared with the female control group at a specific time point, the only considerable difference was noted at 8 WPI (Figure S4).

## Discussion

Based on its previous successful use against *F. hepatica* infection in rats (11), a lettuce-based edible vaccine expressing CPFhW fused to an HBcAg carrier was tested in the natural hosts (sheep and cattle). To the best of our knowledge, this study is the first report of the oral vaccination of ruminants with a plant-produced liver fluke antigen. Previously, we have demonstrated that CPFhW in both protein and cDNA form induced varied and significant levels of protection in rats (17). Moreover, CPFhW administration in the form of inclusion bodies contributed to significant protection in rats (18) and yielded mixed success in sheep and cattle (7). Both the bioinformatics analysis and substrate specificity assays indicate that CPFhW can be classified as a cathepsin L1 (18); the protease is known to play pivotal roles in liver migration, tissue feeding and blood digestion (3).

Previously we showed that feeding mice lettuce expressing CPFhW induced specific and detectable antibody responses and had acceptable safety in the recipient animals (12). Furthermore, lettuce-derived CPFhW tested as a vaccine antigen in rats provided substantial protection in the range of 50–65.4% after subsequent challenge (11). Once we verified that lettuce-derived CPFhW induced both humoral and cellular responses and had immunoprotective potential in laboratory animals, preliminary trials including sheep and cattle were considered viable.

Since vaccines containing single proteins may not be sufficiently immunogenic, the use of non-infectious carriers to deliver relevant epitopes to the immune system is recommended to enhance the vaccine efficacy (19). Here, HBsAg carrier was used as numerous studies have already demonstrated its strong inherent immunogenicity (19–21). Our previous studies also support the use of this carrier system as we have reported that rat immunization with lettuce-derived CPFhW fused to HBcAg resulted in protection against the challenge with *F. hepatica* that was superior to the protection provided by the non-fused antigen (11). Moreover, immunodominant internal site of HBcAg (c/e1 epitope) was selected as the preferred position for CPFhW insertion within the HBcAg protein (11) and CPFhW flanked by flexible Gly-rich linkers inserted into the c/e1 epitope of the HBcAg carrier for oral immunization of sheep and cattle was used. Further, a truncated HBcAg lacking the C-terminal protamine-like arginine-rich domain was used. The domain is dispensable for the correct folding and assembly of HBcAg, although the lack of this domain markedly increases the concentration of dimers needed to drive HBcAg particle assembly (22). The data from the literature suggest that the particulate structure of HBcAg is responsible for the enhanced immunogenicity of this protein and the antigens fused to it.

Calves and lambs fed freeze-dried lettuce expressing CPFhW fused to HBcAg on two occasions showed reductions in worm burdens compared with the control animals that received the same amount of freeze-dried unmodified lettuce. For the vaccinated cattle, a reduction in liver fluke recovery of 56% was reported; however, when the data were analyzed by sex, reductions of 46 and 68% were observed for the males and females, respectively. Contrarily, the male lambs were better protected by vaccination (55%) than their female counterparts (20%); the overall protection from infection in sheep was estimated to be 36%. Thus, cattle vaccinated with a lettuce-based edible vaccine expressing CPFhW were better protected from *F. hepatica* infection than sheep. The level of protection observed in the present vaccine study was within the range seen in cattle and sheep experiments performed to date with various FhCL1 preparations delivered via standard routes (4, 6, 23–25), however, in contrast to the vaccination experiments reported to date, no adjuvants were used during the present vaccination trials.

Here, the liver pathology was mitigated primarily in the female cattle and male sheep vaccinated with lettuce-derived CPFhW fused to the HBcAg carrier, since the protected animals showed reduced liver damage based on the liver damage scores. At 12 WPI, the lowest GGT activities were detected among the CPFhW-vaccinated female cattle and male sheep, which provided biochemical confirmation of the reduced liver damage assessed by visual examination (26).

Reductions in the worm burdens and liver damage were accompanied by significant reductions in the fecal egg outputs of 50 and 34% in the cattle and sheep, respectively. Piacenza et al. (6) also observed a significant reduction in the fluke egg output of 71% in sheep vaccinated with CL1. The enzyme used in the present vaccine study (CPFhW) previously showed potential for reducing *F. hepatica* fecundity (7). Here, we demonstrated that flukes developed in orally vaccinated cattle showed reduced fecundity when judged by egg numbers per gram of feces; this effect was not seen among the vaccinated sheep. Additionally, we found previously that fluke eggs obtained from vaccinated calves possessed reduced hatchability (27). The effects of the vaccine on egg production and the “hatch rate” may be mediated by specific antibodies that inhibit parasite feeding by blocking cathepsin L activity, thereby preventing the acquisition of amino acids needed for the synthesis of egg proteins (28). Thus, the oral vaccine containing CPFhW not only reduces the worm burdens but also influences the extent of pasture contamination and hence disease transmission. These results are similar to the findings reported by Dalton et al. (4), who vaccinated cattle using FhCL1, FhCL2, and hemoglobin. Additionally, the oral CPFhW vaccine had an adverse effect on parasite size in the vaccinated cattle but not in the vaccinated sheep. The highest proportion of flukes shorter than 10 mm and the lowest proportion of flukes longer than 20 mm were noted in the vaccinated female cattle.

In the present study, the experimental groups consisted of animals of both sexes; however, when the data was analyzed by sex, clear differences in liver fluke recovery were noted. Most articles published to date have failed to analyse sex differences during fasciolosis with the majority of animal research performed on males only, although female farm animals are often more economically important (e.g., dairy cattle). Since males and females significantly differ in their responses to antigenic challenge, analyzing the sexes together or extrapolating outcome data from one sex to another may lead to erroneous conclusions that can have serious implications for drug and vaccine studies. Indeed, sex effects have been reported for many commercially available vaccines in use in humans (29). Our previous experiments regarding fasciolosis also showed variable vaccine efficacies in male and female hosts (7, 30–33). Here, we reported that female cattle were better protected by vaccination than male cattle. Conversely, orally vaccinated male lambs possessed lower fluke burdens than vaccinated females, although the differences were not significant. Since the group size was small, use of groups with higher numbers of males and females and more detailed immunological studies are needed to verify and elucidate these phenomena. Mechanisms of sex-biased differences have to be explored in further studies. Due to the effects of sex exclusion in vaccine studies, we most likely lack important scientific information concerning how to provide optimal disease management for both sexes.

In addition to sex influence, the host species was a significant criterion that altered the vaccination outcomes in the present study. Cattle responses evoked by oral vaccination were more successful in reducing worm burdens, whereas the overall protection observed in the sheep was moderate. Sheep have been shown to be highly susceptible to *F. hepatica* infection, since they do not develop the marked fibrosis of the liver tissue or bile duct calcification that is observed in cattle (34). Additionally, flukes developing in sheep grow faster, more uniformly and to a greater size than flukes developing in cattle (35). As was recently suggested, vaccine targets may potentially be the same for both species; however, the vaccine formulations, delivery regimes and administration methods should be adjusted discretely for cattle and sheep to mount the most efficacious immune response (3). Nevertheless, delineating immune differences in the course of *F. hepatica* infection between cattle and sheep is fundamental for further research aimed at vaccine development.

Previous investigations of immune responses in cattle and sheep to *F. hepatica* infection have shown that these hosts mainly produce IgG1 antibodies associated with a Th2 response (14, 36). Here, we observed increased specific IgG titres in serum samples collected from the orally vaccinated cattle and sheep from 6 to 12 WPI. Moreover, the comparison of IgG responses between the male and female animals showed a sex difference, with the female cattle and male sheep having higher IgG titres than their counterparts. This result correlates with the higher reductions in liver fluke recoveries in these groups. Similarly, a positive correlation occurred between protection and antibody titres in cathepsin L-vaccinated calves, with high total antibody titres correlated with lower fluke burdens (37).

One could anticipate that vaccination would elicit increased titres of CPFhW specific antibodies after first and second immunization. Surprisingly, there is no clear evidence that vaccine has raised specific antibody response to CPFhW in the animals before challenge. The vaccine effect was seen only from 6 WPI up to the end of the experiment. Further, since CPFhW is being classified as CL1 and those proteases are not expressed/secreted by early-stages of liver fluke, it explains why low IgG levels were observed at 0–4 WPI. Previous analysis of CL1 specific antibodies in infected cattle revealed that these molecules do not appear in serum until 4–5 WPI (38). Also our findings from the presented study confirm this trend.

As CL1s have well-documented roles in digestion, migration and immune evasion (38), neutralizing cathepsins' activities by specific antibodies interferes with the parasite feeding, movement and immune evasion tactics, thereby reducing worm burden and liver pathology. Moreover, previous vaccine studies indicate that vaccination with *F. hepatica* CL1 affects egg production and eggs' hatch rate by a mechanism involving anti-CL1 antibodies (38). Presumably, by neutralizing CL1 activity parasite feeding is impaired, thereby preventing the acquisition of amino acids needed for the synthesis of egg proteins. Also, it was demonstrated that anti-CL1 antibodies by blocking cathepsin activity, hamper antibody mediated eosinophil attachment to the liver fluke (39).

The vast majority of vaccines against *F. hepatica* infection have been delivered with a variety of adjuvants by the intramuscular or subcutaneous routes. Although this approach is known to induce clear systemic responses, it is ineffective at eliciting protective immunity at mucosal surfaces. The efficacy of injectable vaccines against pathogens that initiate infection at mucosal surfaces has been generally unsatisfactory (40). When considering parasites such as *F. hepatica*, the best target for a vaccine seems to be a mucosal surface itself, since liver flukes initiate infection from intestinal mucosa penetration, and control over the early immune responses is crucial for the pathogen's ability to establish an infection (41). Hence, stimulation of mucosal immunity may significantly improve the vaccine efficacy, and indeed oral or intranasal use of *F. hepatica* cathepsin proteases has been shown to be beneficial against rat and ruminant fasciolosis (7, 17, 18). Here, an orally administered edible vaccine also induced considerable protection. However, the actual reduction in fluke numbers in vaccinated animals was comparable to previous trials with FhCL preparations delivered via standard routes in ruminants. Still, further studies are required to characterize immunity at the vaccine delivery site to determine if lettuce-derived FhPCW generated a mucosal response and if protective effect of the edible vaccine is a result of effective priming at the gut level. The issue of mucosal immunity remains to be addressed in future studies. Here, we failed to demonstrate specific IgA responses in sera collected from experimental animals.

A key feature of plant-made vaccines is that the vaccine antigens are bioencapsulated by plant cell walls, which protects them from degradation in the acidic environment of the stomach. Once the vaccine reaches the gut, commensal microbes digest the cell walls, and the vaccine antigens are released in their biologically active forms for processing by immune cells. As a consequence, both mucosal and systemic immunity are stimulated (42). Antigens of human and farm animal pathogens have been successfully expressed in plants and tested in numerous vaccine trials, which have reported the immunogenicity of orally delivered plant-made vaccines (9). Initially, the issue with ruminants was whether their complex four-compartment stomachs might affect the vaccine prior to its transit to the intestine. As opposed to monogastric animals, in ruminants degradation of plant cell wall begins in stomach before plant material reaches the intestine, and a significant portion of the vaccine protein is likely degraded in the rumen. Still, a portion of the protein is released in the intestine, taken by M cells that pass the vaccine antigen to macrophages and B cells. The latter cells activate T cells to initiate the immune response to the plant-made vaccine. Alternatively, other scenario of immune response induction that exploits the process of rumination is possible. During cudding the vaccine antigen may be exposed to the pharyngeal lymphoid tissue and consequently induce memory cells that migrate to the intestine, priming that site for subsequent natural exposure (43). It has been suggested that pharyngeal exposure primes the intestine for an anamnestic response against the pathogen, rather than results in production of antibodies at levels observed for antigens delivered by injection (43). Such a scenario may explain why low antibody levels were reported in pre-challenge period in our study. Further, also other studies have demonstrated plant-made vaccine efficacy in ruminating animals and have reported specific mucosal and systemic responses (44–47). Thus, oral delivery of plant-made vaccines is a viable alternative in ruminant vaccination.

Oral delivery of vaccine in plant cells may result in immunosupression, hence oral tolerance is a potential concern for the production of plant-based vaccines (48). Initial studies have demonstrated limited/low success of oral priming (49–51). Some authors have suggested a vaccination regimen that combines a plant-produced antigen as an oral booster after a primary intramuscular injection to improve the vaccine efficacy. Indeed, enhanced immune responses have been already demonstrated in studies using a scheme comprising parenteral priming with conventional antigen and oral boosting (52–55). Still, repeated or continuous exposure is usually necessary to trigger immunosuppresion as the induction of oral tolerance is both time- and dose-dependent. In general, the antigen dose necessary to induce protection is smaller than the dose required to induce tolerance (56). In our previous study on the oral vaccination of rats against *F. hepatica*, we observed that two oral doses of 300 μg of *E. coli*-expressed CPFhW protein per animal induced 79% protection (18). Here, we administered similar doses of proteins to lambs, which possessed an ~100 times higher body mass. Additionally, CPFhW antigen administered in plants was expressed as a fusion protein of CPFhW inserted into an HBcAg carrier to potentiate the immune response of the target antigen to which it was fused. This approach allowed a low dose of the antigen to be used and thereby reduced the risk of developing immunogenic tolerance. However, other vaccination regimens or adjuvants may need to be included to enhance vaccine immunogenicity in future studies.

In conclusion, the present study demonstrates that oral immunization with a plant-made and plant-delivered vaccine expressing CPFhW fused to an HBcAg carrier is a viable alternative to vaccines delivered parenterally. Improved understanding of mucosal immune effector mechanisms in relevant target host species is a prerequisite for further studies aimed at developing new vaccine strategies. Moreover, sex-related differences in vaccine efficacy must be elucidated; this issue requires more detailed study, because inattention to sex as a research criterion has most likely contributed in part to the lack of success in anti-*F. hepatica* vaccine development. Thus, future studies addressing these problems are highly anticipated.

## Author contributions

AL and HW contributed to the conception and the design of the study. AW, MK-B, KB, MK, and LJ performed the investigation. AW wrote the first draft of the manuscript and performed the statistical analysis. AW, KB, and MK-B wrote sections of the manuscript. All authors read and approved the submitted version.

### Conflict of interest statement

The authors declare that the research was conducted in the absence of any commercial or financial relationships that could be construed as a potential conflict of interest.

## Supplementary material

Table S1 Sex-related analysis of liver fluke recoveries in infected cattle and sheep at 12 WPI. ^*^Denotes a significant difference compared to respective control group (*p* < 0.05).

Table S2 Numbers of fluke eggs found in the infected cattle and sheep of both sexes at necropsy (12 WPI). ^*^Denotes a significant difference compared to respective control group (*p* < 0.05).

Table S3 Sex-related analysis of vaccination influence on fluke body size.

Table S4 Sex-related analysis of liver damage scores. ^*^Denotes a significant difference compared to respective control group (*p* < 0.05).

Figure S1 Sex-related analysis of liver enzymes activities in sera collected from experimental animals throughout the study. ^*^Represents statistically significant differences (*p* < 0.05).

Figure S2 Sex-related analysis of eosinophil and total white blood cell (WBC) counts in blood samples collected from experimental animals. ^*^Represents statistically significant differences (*p* < 0.05).

Figure S3 Sex-related analysis of CD4 and CD8 T cell counts in blood samples collected from experimental animals. ^*^Represents statistically significant differences (*p* < 0.05).

Figure S4 Sex-related analysis of total IgG levels in sera collected from experimental animals. ^*^Represents statistically significant differences (*p* < 0.05).
