# Supplementary material for: A Preliminary Study of a Lettuce-Based Edible Vaccine Expressing the Cysteine Proteinase of Fasciola hepatica for Fasciolosis Control in Livestock
Source: Front Immunol. 2018 Nov 13;9:2592. doi: 10.3389/fimmu.2018.02592 (PMC6244665; doi:10.3389/fimmu.2018.02592)
Supplement: Table S1 — Sex-related analysis of liver fluke recoveries in infected cattle and sheep at 12 WPI. *Denotes a significant difference compared to respective control group (p < 0.05). [file Table_1.DOC]

**Table S1.** **Sex-related analysis of liver fluke recoveries in infected cattle and sheep at 12 WPI.**

| **group** | **sex** | **liver fluke counts** | **total liver**  **fluke counts** | **mean liver fluke count ± SD** | **reduction in liver fluke burden** |
| --- | --- | --- | --- | --- | --- |
| cattle fed with CPFhW/lettuce | ♂  ♀ | 36, 39, 42  16, 20, 24 | 117  60 | 39.0±3.0*  20.0±4.0* | 45.8%  68.1% |
| cattle fed with control lettuce | ♂  ♀ | 56, 59, 101  48, 59, 81 | 216  188 | 72.0±25.1  62.7±16.8 |  |
| sheep fed with CPFhW/lettuce | ♂  ♀ | 27, 36, 44  25, 80, 138 | 107  243 | 35.7±8.5*  81.0±56.5 | 54.7%  20.3% |
| sheep fed with control lettuce | ♂  ♀ | 65, 79, 92  84, 94, 127 | 236  305 | 78.7±13.5  101.7±22.5 |  |

* denotes a significant difference compared to respective control group (p<0.05)
