# Supplementary material for: A Preliminary Study of a Lettuce-Based Edible Vaccine Expressing the Cysteine Proteinase of Fasciola hepatica for Fasciolosis Control in Livestock
Source: Front Immunol. 2018 Nov 13;9:2592. doi: 10.3389/fimmu.2018.02592 (PMC6244665; doi:10.3389/fimmu.2018.02592)
Supplement: Table S2 — Numbers of fluke eggs found in the infected cattle and sheep of both sexes at necropsy (12 WPI). *Denotes a significant difference compared to respective control group (p < 0.05). [file Table_2.DOC]

**Table S2.** **Numbers of fluke eggs found in the infected cattle and sheep of both sexes at necropsy (12 WPI).**

| **group** | **sex** | **no. of eggs  per gram of feces** | **no. of fluke eggs  in the gall bladders** |
| --- | --- | --- | --- |
| cattle fed with CPFhW/lettuce | ♂  ♀ | 2.67±0.58*  1.33±0.58* | 45,213±20,839  33,213±12,326* |
| cattle fed with  control lettuce | ♂  ♀ | 4.33±0.58  3.67±0.58 | 69,540±16,329  71,947±12,359 |
| sheep fed with CPFhW/lettuce | ♂  ♀ | 2.33±1.53  2.67±0.58 | 28,153±40,688  49,091±23,638 |
| sheep fed with  control lettuce | ♂  ♀ | 4.00±1.00  3.67±2.08 | 336,788±274,497  279,565±200,810 |

* denotes a significant difference compared to respective control group (p<0.05)
