# Supplementary material for: A Preliminary Study of a Lettuce-Based Edible Vaccine Expressing the Cysteine Proteinase of Fasciola hepatica for Fasciolosis Control in Livestock
Source: Front Immunol. 2018 Nov 13;9:2592. doi: 10.3389/fimmu.2018.02592 (PMC6244665; doi:10.3389/fimmu.2018.02592)
Supplement: Table S3 — Sex-related analysis of vaccination influence on fluke body size. [file Table_3.DOC]

**Table S3.** **Sex-related analysis of vaccination influence on fluke body size.**

| **group** | **sex** | **percentage [%] of flukes with body size [mm]** | | |
| --- | --- | --- | --- | --- |
|  |  | **<10** | **10-20** | **>20** |
| cattle fed with CPFhW/lettuce | ♂  ♀ | 22  28 | 52  50 | 26  22 |
| cattle fed with control lettuce | ♂  ♀ | 14  8 | 69  61 | 17  31 |
| sheep fed with CPFhW/lettuce | ♂  ♀ | 24  18 | 64  58 | 12  24 |
| sheep fed with control lettuce | ♂  ♀ | 18  15 | 63  62 | 19  23 |
