# Supplementary material for: A Preliminary Study of a Lettuce-Based Edible Vaccine Expressing the Cysteine Proteinase of Fasciola hepatica for Fasciolosis Control in Livestock
Source: Front Immunol. 2018 Nov 13;9:2592. doi: 10.3389/fimmu.2018.02592 (PMC6244665; doi:10.3389/fimmu.2018.02592)
Supplement: Table S4 — Sex-related analysis of liver damage scores. *Denotes a significant difference compared to respective control group (p < 0.05). [file Table_4.DOC]

**Table S4.** **Sex-related analysis of liver damage scores.**

| **group** | **sex** | **liver damage scores** |
| --- | --- | --- |
| cattle fed with CPFhW/lettuce | ♂  ♀ | 3.00±1.00  2.33±0.58* |
| cattle fed with control lettuce | ♂  ♀ | 4.00±1.00  3.67±0.58 |
| sheep fed with CPFhW/lettuce | ♂  ♀ | 3.33±0.58*  4.00±0.00 |
| sheep fed with control lettuce | ♂  ♀ | 4.67±0.58  4.33±0.58 |

* denotes a significant difference compared to respective control group (p<0.05)
