# Supplementary figures and images for: A Preliminary Study of a Lettuce-Based Edible Vaccine Expressing the Cysteine Proteinase of Fasciola hepatica for Fasciolosis Control in Livestock
Source: Front Immunol. 2018 Nov 13;9:2592. doi: 10.3389/fimmu.2018.02592 (PMC6244665; doi:10.3389/fimmu.2018.02592)

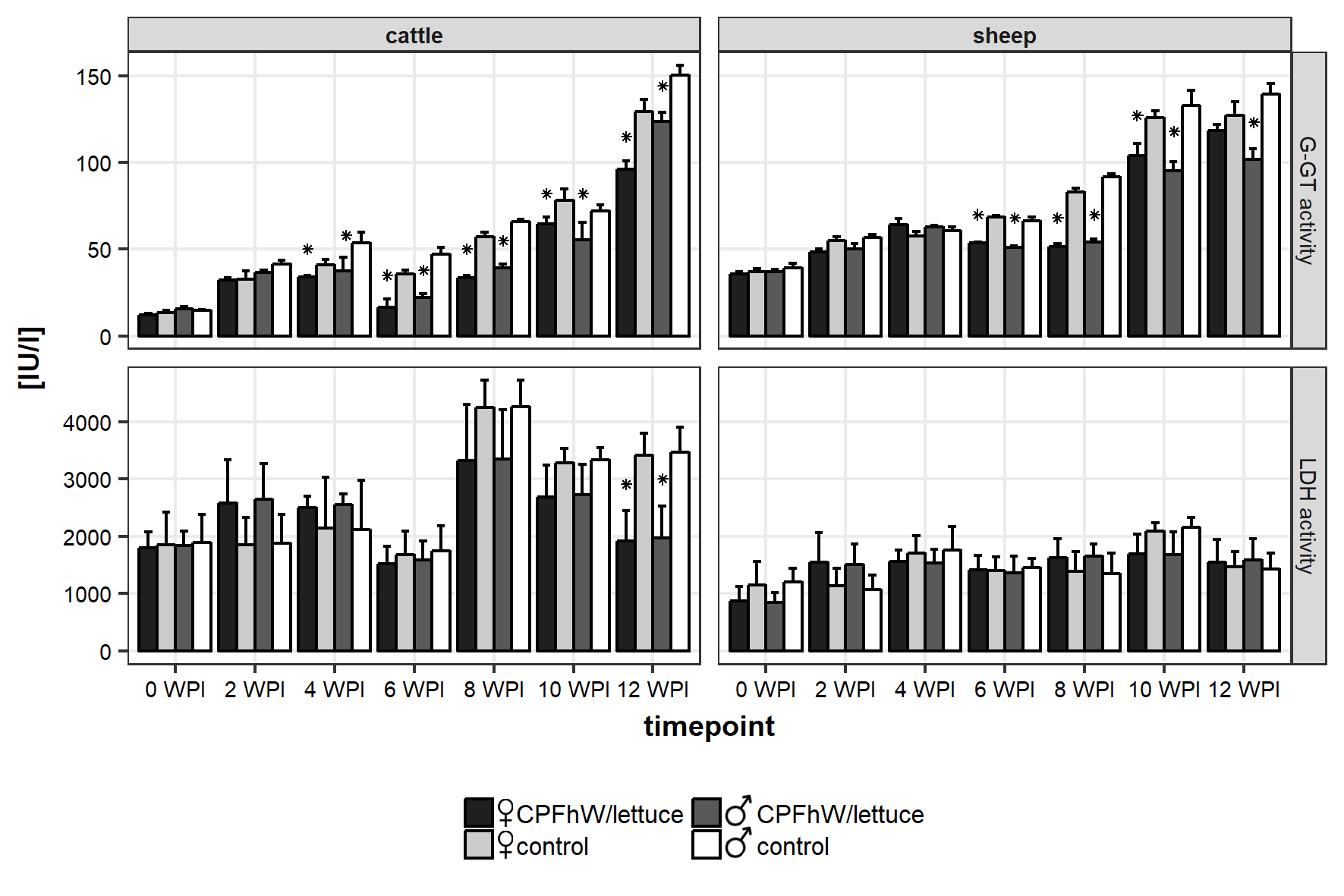

Supplement: Figure S1 — Sex-related analysis of liver enzymes activities in sera collected from experimental animals throughout the study. *Represents statistically significant differences (p < 0.05). [file Image_1.TIF]

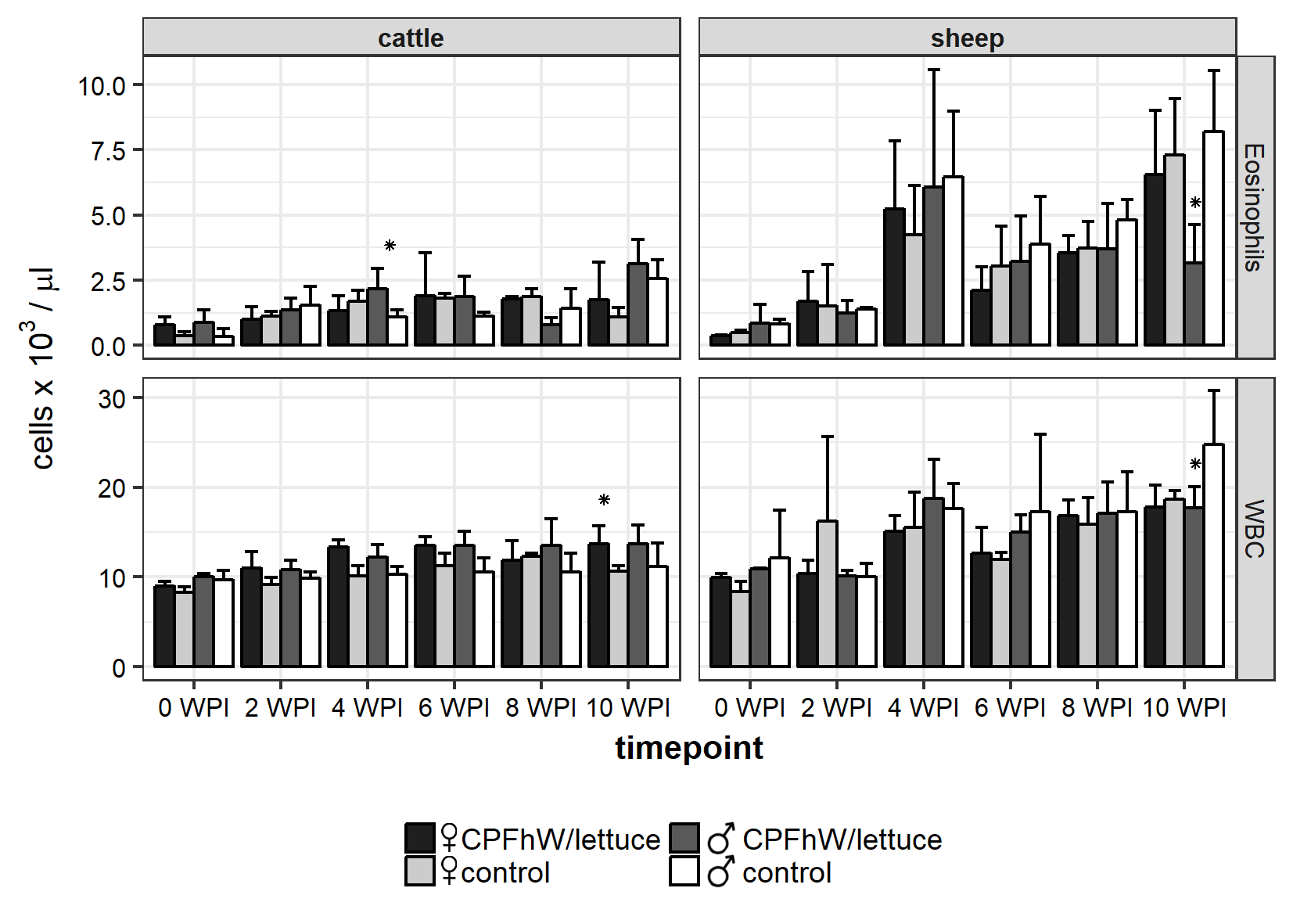

Supplement: Figure S2 — Sex-related analysis of eosinophil and total white blood cell (WBC) counts in blood samples collected from experimental animals. *Represents statistically significant differences (p < 0.05). [file Image_2.TIF]

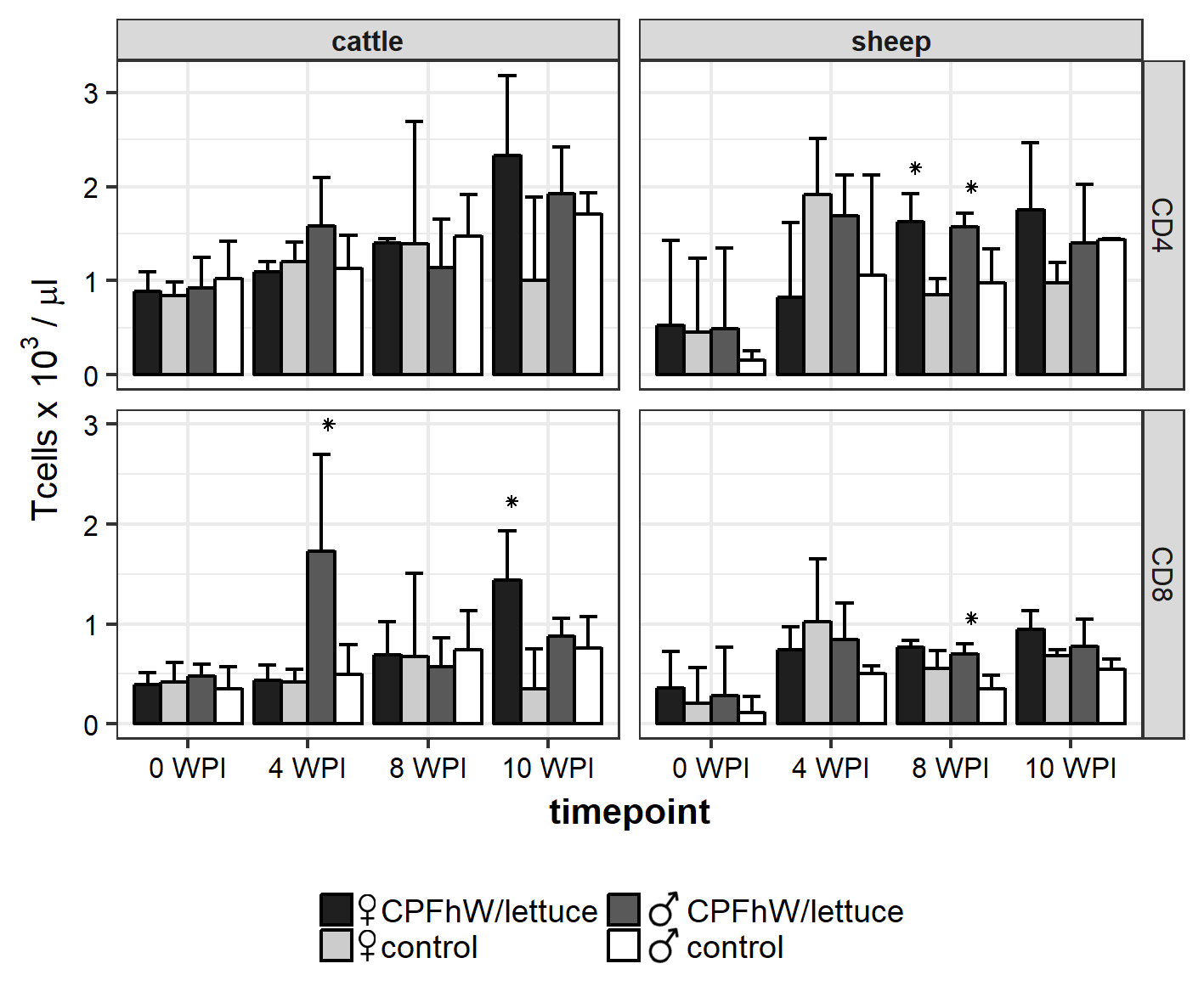

Supplement: Figure S3 — Sex-related analysis of CD4 and CD8 T cell counts in blood samples collected from experimental animals. *Represents statistically significant differences (p < 0.05). [file Image_3.TIF]

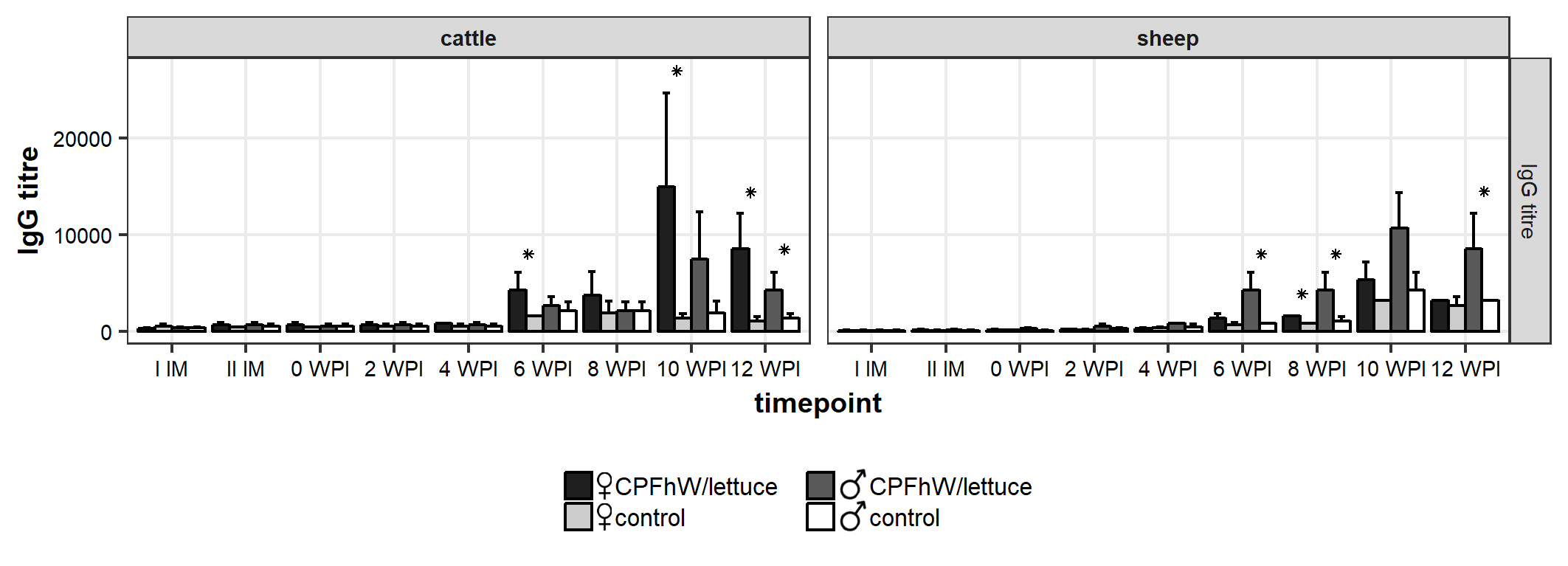

Supplement: Figure S4 — Sex-related analysis of total IgG levels in sera collected from experimental animals. *Represents statistically significant differences (p < 0.05). [file Image_4.TIF]
